# Behind the Dizziness: A Clinical Journey to Wallenberg Syndrome

**DOI:** 10.7759/cureus.95227

**Published:** 2025-10-23

**Authors:** Maaz Khalid, Madeena Mahmood

**Affiliations:** 1 Internal Medicine, The Royal Wolverhampton NHS Trust, Wolverhampton, GBR; 2 General Medicine, Mersey and West Lancashire Teaching Hospitals NHS Trust, Liverpool, GBR

**Keywords:** acute ischaemic stroke, dual anti-platelet therapy, dw-mri, horner’s syndrome, lateral medullary syndrome (wallenberg syndrome)

## Abstract

Wallenberg syndrome, also referred to as lateral medullary syndrome or posterior inferior cerebellar artery (PICA) syndrome, is a rare neurological disorder most often caused by occlusion of the PICA. It leads to infarction of the lateral medulla oblongata and presents with a range of symptoms, including vertigo, ataxia, cranial nerve deficits, and sensory disturbances. We present the case of a 41-year-old female who initially attended the emergency department (ED) with headache, nausea, and vomiting. She was treated for a urinary tract infection (UTI) with associated urinary retention. However, shortly after being discharged, she collapsed within the hospital grounds. Her medical history included hypertension and type 2 diabetes mellitus (T2DM). A computed tomography (CT) head scan showed no acute findings, but a subsequent magnetic resonance imaging (MRI) confirmed an acute left lateral medullary infarct, establishing the diagnosis of Wallenberg syndrome. This atypical presentation, which caused the misdiagnosis, can have a serious effect on the treatment of a stroke and the important decision to thrombolyse or not. This delay in treatment could cause long-term damage to the patient by affecting their day-to-day life.

## Introduction

Wallenberg syndrome is also known as lateral medullary and posterior inferior cerebellar artery (PICA) syndrome. This is a rare neurological condition most commonly resulting from occlusion of the PICA [1]. This syndrome is characterized by a variety of symptoms, including vertigo, ataxia, sensory deficits, and cranial nerve abnormalities, arising from infarction of the lateral medulla oblongata. This rare neurological condition was first described in 1808 by Gaspard Vieusseux; however, it was further detailed by Adolf Wallenberg in 1895 [2].

The PICA arises from the vertebral artery below the vertebrobasilar junction. The PICA supplies the medulla, the choroid plexus, and the tela choroidea (a thin vascular layer of pia mater) of the fourth ventricle. This syndrome is caused by an atherothrombotic occlusion of the vertebral artery. A typical patient with lateral medullary syndrome or Wallenberg syndrome is usually an elderly patient with vascular risk factors such as hypertension and type 2 diabetes mellitus. Other risk factors, such as smoking and hypercholesterolemia, are also commonly seen. Symptoms include dizziness with vertigo, loss of balance with gait instability, dysphonia, and dysphagia. There is usually minimal or no weakness; hence, it can often be misdiagnosed or missed [3]. The case being presented is an example of how Wallenberg syndrome can be misdiagnosed. The patient had an unusual symptom sequence, which led to the diagnosis, as she had initially presented with symptoms of a urinary tract infection (UTI) and had been treated accordingly. The collapse on the hospital grounds was the turning point in the patient's journey to being diagnosed with Wallenberg syndrome.

Diagnosis of Wallenberg syndrome requires a comprehensive history and a thorough neurological examination. MRI with diffusion-weighted imaging (DWI) is the best diagnostic test for confirming the location of the infarct. Horner's syndrome is a cluster of signs resulting from the interruption of sympathetic innervation to the eye and ocular adnexae [4]. Typical signs of Horner's syndrome include a triad of ipsilateral ptosis, pupillary miosis, and facial anhidrosis [5]. The condition itself usually does not cause significant visual symptoms, but it is an important clinical sign indicating involvement of the oculosympathetic pathway. The pathway of the oculosympathetic system originates in the hypothalamus and travels through the brainstem and spinal cord. The second-order neuron then ascends through the thorax and neck and synapses at the superior cervical ganglion (SCG). Finally, the third-order neuron arises from the SCG to the eye, innervating the iris dilator muscle. Due to the long and complex pathway, this becomes vulnerable to any pathology involving the head, chest, or neck.

This case is significant because it highlights the importance of maintaining a high index of suspicion for central nervous system pathology in patients presenting with atypical or nonspecific symptoms. The initial misdiagnosis of a UTI underscores how subtle or misleading early clinical features of Wallenberg syndrome can be, particularly when classic neurological signs are absent. Early recognition and accurate diagnosis are crucial, as prompt neuroimaging and targeted management can significantly improve outcomes. This case serves as a reminder to clinicians to consider posterior circulation strokes in the differential diagnosis when patients present with unexplained dizziness, nausea, or sensory changes, even in the presence of seemingly unrelated findings.

## Case presentation

A 41-year-old female presented to the emergency department with a headache, nausea, and vomiting. At this hospital, she was initially managed and treated as having a UTI with urinary retention and was found to be constipated. In the emergency department, she was given a phosphate enema and catheterized. The following day, she was discharged on antiemetics and antibiotics. She was later found collapsed on the hospital grounds. Unfortunately, the time between discharge and the collapse is unknown, but it is thought that she was walking out of the hospital to the exit when she collapsed. After regaining consciousness, she complained of a gradual-onset left frontal headache, where the pain had radiated to her eyes. She was unable to bear weight and exhibited ataxia; additionally, she experienced chest pain and blurred vision during her collapse. Neurological examination revealed GCS 15/15, a left nasolabial fold droop, and power was maintained on the right, but there was 4/5 power and reduced sensation in the left upper and lower limbs.

Upon examination, due to the new-onset neurological deficit, the National Institutes of Health Stroke Scale (NIHSS) score was found to be 10. The NIHSS score of 10 indicates that the stroke was of a moderate nature. There was a withdrawal of the left plantar and an absent right plantar. Initially, the differential diagnosis was meningoencephalitis, spinal infection, or lupus, with the possibility of being a migraine and UTI. Due to the previous UTI, she was started on cefalexin to treat her symptoms as she awaited the scan. Furthermore, a CT angiogram showed a normal vertebrobasilar arterial system, with normal appearance of the vertebral, basilar, and both posterior cerebral arteries. On admission, her blood results were unremarkable (Table 1).

**Table 1:** Blood results with reference range on admission, which were unremarkable EPI eGFR = Estimated Glomerular Filtration Rate; CRP = C-Reactive Protein; Hb = Hemoglobin; WBC = White Blood Cells; PLTS = Platelets; RBC = Red Blood Cells; Hct = Hematocrit; MCV = Mean Corpuscular Volume; MCH = Mean Corpuscular Hemoglobin; MCHC = Mean Corpuscular Hemoglobin Concentration; NRBC = Nucleated Red Blood Cell

Laboratory Test	Results	Reference Range
Sodium	140	133–146 mmol/L
Potassium	4.3	3.5–5.3 mmol/L
Urea	2.5	2.5–7.8 mmol/L
Creatine	67	50–98 µmol/L
EPI eGFR	>90	>59 mL/min/1.73 m^2^
CRP	1	0.0–5.0 mg/L
Hb	140	115–165 g/L
WBC	11.30	4.0–11.0 x10^9^/L
PLTS	226	150–450 x10^9^/L
RBC	4.48	3.8–5.8 x10^12^/L
Hct	0.392	0.37–0.47 L/L
MCV	87.5	80–100 fL
MCH	31.3	27–32 pg
MCHC	357	320–360 g/L
NRBC	0.00	0–0.2 x10^9^/L
Neutrophils	6.92	2–7.5 x10^9^/L
Lymphocytes	3.49	1.5–4.5 x10^9^/L
Monocytes	0.69	0.2–0.8 x10^9^/L
Eosinophils	0.14	0.0–0.4 x10^9^/L
Basophils	0.07	0.0–0.1 x10^9^/L

**Figure 1:**
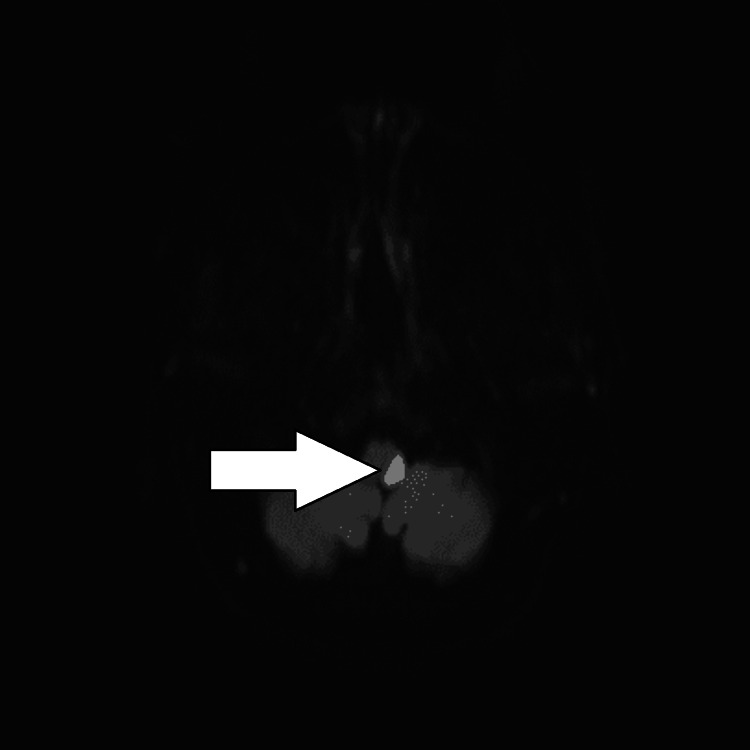
MRI with DWI There is a restricted area of diffusion in the medulla on the left side, which is consistent with an acute medullary infarct on the left side. MRI with DWI remains a key modality in the diagnosis of a medullary infarct. MRI = Magnetic Resonance Imaging; DWI = Diffusion-Weighted Imaging

**Figure 2:**
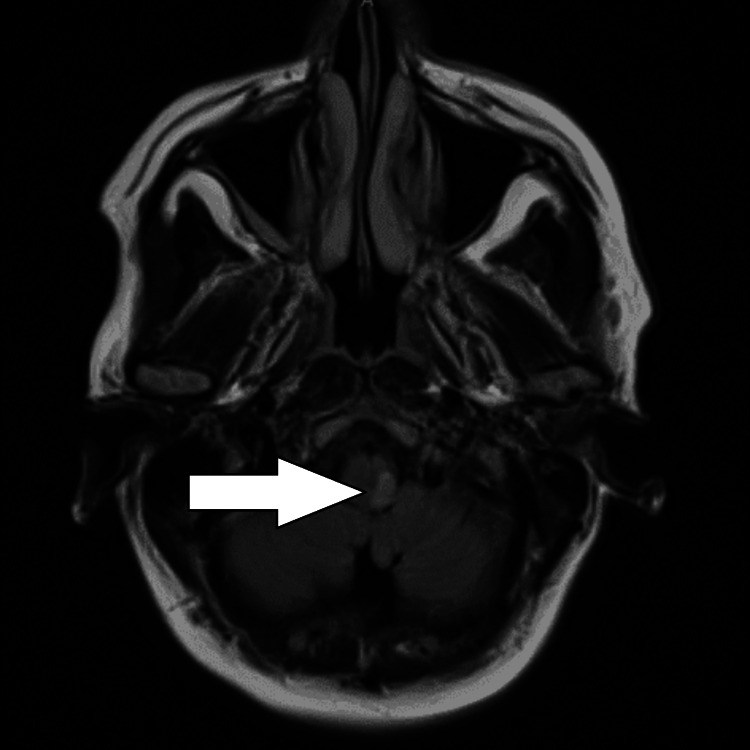
MRI T2 FLAIR The restricted area of diffusion in the medulla on the left side is also visible on the T2 FLAIR, which is consistent with an acute medullary infarct on the left side. The hyperintensity seen in the T2 FLAIR is known to be associated with stenosis and occlusion of vessels and the attendant flow. This is indicated by the arrow in the figure. MRI = Magnetic Resonance Imaging; FLAIR = Fluid-Attenuated Inversion Recovery

She underwent a CT scan and an MRI to help diagnose her symptoms. The CT of the head was nil acute, and to rule out any spinal differentials, an MRI of the spine was performed but showed no significant abnormalities. An MRI of the head was then performed a day later, which showed an acute left medullary infarct on the DWI (Figure 1) and FLAIR (fluid-attenuated inversion recovery) T2 (Figure 2) sequencing. She was immediately started on aspirin 300 mg, clopidogrel 75 mg, atorvastatin 40 mg OD (once daily), and lansoprazole 30 mg OD as gastric protection. Early initiation of dual antiplatelet therapy (DAPT) is supported by evidence from randomized clinical trials, such as CHANCE and POINT, which demonstrated improved outcomes in patients with acute ischemic stroke. The full workup of a stroke diagnosis continued with a 24-hour tape, which revealed no significant findings. Her echocardiogram showed normal bi-ventricular size and systolic function. The LV diastolic function was normal, and there was no significant valve pathology. Due to unremarkable findings on both the 24-hour tape and ECHO, an outpatient 72-hour tape was requested, which showed no significant findings.

She consistently had episodes of vomiting after eating, which was due to reflux, and continued her lansoprazole with Peptac as and when needed. An ongoing pain in the left side of her body from the head to the chest and to the left foot, for which an ECG (electrocardiogram) was done to rule out any cardiac abnormalities; however, the ECG showed a normal sinus rhythm with no abnormalities. Towards the end of her hospital journey, she suddenly had a temperature spike of 39℃, and the blood test (Table 2) was suspicious for neutropenic sepsis. However, a urine MCS (microscopy, culture, and sensitivity) showed E. coli (Escherichia coli), and she was started on antibiotics for further treatment.

**Table 2:** Blood results with reference ranges showing raised CRP but low neutrophils showing possible neutropenic sepsis EPI eGFR = Estimated Glomerular Filtration Rate; CRP = C-Reactive Protein; Hb = Hemoglobin; WBC = White Blood Cells; PLTS = Platelets; RBC = Red Blood Cells; Hct = Hematocrit; MCV = Mean Corpuscular Volume; MCH = Mean Corpuscular Hemoglobin; MCHC = Mean Corpuscular Hemoglobin Concentration; NRBC = Nucleated Red Blood Cell

Laboratory Test	Results	Reference Range
Sodium	133	133–146 mmol/L
Potassium	4.2	3.5–5.3 mmol/L
Urea	3.5	2.5–7.8 mmol/L
Creatine	63	50–98 μmol/L
EPI eGFR	>90	>59 mL/min/1.73 m^2^
CRP	208	0.0–5.0 mg/L
Hb	120	115–165 g/L
WBC	4.60	4.0–11.0 x10^9^/L
PLTS	369	150–450 x10^9^/L
RBC	3.96	3.8–5.8 x10^12^/L
Hct	0.322	0.37–0.47 L/L
MCV	81.3	80–100 fL
MCH	30.3	27–32 pg
MCHC	373	320–360 g/L
NRBC	0.00	0–0.2 x10^9^/L
Neutrophils	0.32	2–7.5 x10^9^/L
Lymphocytes	2.31	1.5–4.5 x10^9^/L
Monocytes	1.88	0.2–0.8 x10^9^/L
Eosinophils	0.04	0.0–0.4 x10^9^/L
Basophils	0.05	0.0–0.1 x10^9^/L

She was transferred to a rehabilitation hospital for further physiotherapy, as she remained ataxic and was unable to mobilize independently. She progressed well with therapy and started mobilizing with a frame; however, the ongoing pain in the left side of her body was not resolving. She was on regular paracetamol with codeine as medication when required.

On examination, she was found to have developed left-sided ptosis, miosis, and anhidrosis. And after carrying out a thorough neurological examination, she was diagnosed with Horner's syndrome. The symptoms had been noted during a standard ward round upon re-evaluation of her neurological symptoms, as they had not been improving even four weeks post-stroke. In this case, the treatment for Horner's syndrome involved addressing the stroke that had been diagnosed. In her case, she was already on the pathway of the prevention of a recurrence of a stroke by continuing on lifelong clopidogrel, and the effects of the stroke would improve with time and physiotherapy. She has been discharged back to her own home with a rollator frame and was able to carry out basic activities of daily living herself. Upon discharge, her MRS was re-evaluated and was found to be 1. This indicated that she had no significant disability; however, she still experienced some symptoms but was able to perform all her usual activities. On follow-up, she mentioned that her mobility has been improving and that she is now more independent. She has progressed to using a stick to mobilize and is aiming to be fully independent.

## Discussion

The rare disorder of Wallenberg syndrome was presented with atypical symptoms such as headache, nausea, and vomiting. Although CT imaging of the brain can sometimes show an acute infarct, it is not always necessarily the case. In some cases, such as this patient's, the CT head was negative for acute findings, and the best diagnostic method used was an MRI with DWI and T2 FLAIR sequencing. Considering the patient was outside the thrombolysis window (typically 4.5 hours from onset), the best treatment was oral antiplatelet medications. Early initiation of DAPT is supported by evidence from randomized clinical trials, such as CHANCE and POINT, which demonstrated improved outcomes in patients with acute ischemic stroke. Its implementation has become standard practice in hospital settings, where structured protocols allow for optimization of therapy and promote patient adherence. The importance of early intervention in physiotherapy and occupational therapy plays a key role in aiding patient recovery post-stroke.

The clinical manifestation arising from loss of perfusion to the territory supplied by the PICA is called Wallenberg syndrome; this is often a misdiagnosed subtype of posterior circulation stroke [6]. The typical presentation of a posterior circulation stroke can include dizziness and vertigo, ataxia, cranial nerve deficits, visual disturbances, and dysarthria. A range of additional neurological deficits may occur in varying combinations, largely determined by the extent and precise localization of the infarct within the lateral medulla. Patients may experience a disturbing sensation of being pulled toward the side of the lesion during ambulation, which often reflects involvement of the vestibular and proprioceptive pathways. Similarly, some individuals demonstrate difficulty maintaining an upright seated posture without external support, attributable to impaired integration of cerebellar and vestibular inputs. Visual disturbances, including blurred vision and diplopia, may also be present due to disruption of ocular motor pathways. Furthermore, ipsilateral Horner's syndrome is frequently observed, resulting from interruption of descending sympathetic fibers within the brainstem. With timely diagnosis and treatment, the prognosis can be favorable in many cases. The primary risk factors implicated in arterial atherothrombotic occlusion include chronic hypertension, tobacco use, and diabetes mellitus, all of which contribute significantly to endothelial injury and atherosclerotic disease progression. In younger individuals, however, clinicians must maintain a high index of suspicion for vertebral artery dissection, which may occur secondary to cervical trauma or mechanical manipulation, or in the context of underlying heritable connective tissue disorders such as Ehlers-Danlos syndrome and Marfan syndrome [7].

Posterior circulation strokes such as Wallenberg syndrome remain diagnostically challenging and are frequently underrecognized in the acute setting. Early symptoms, most notably dizziness, nausea, and vomiting, can mimic benign vestibular disorders, contributing to diagnostic delays. This diagnostic pitfall highlights the importance of maintaining a high index of suspicion for brainstem ischemia when evaluating acute vertigo.

In addition to embolic mechanisms, intracranial atherosclerotic stenosis (ICAS) represents an important etiological consideration, particularly in posterior circulation infarctions. Although ICAS was not directly demonstrated in this patient, its potential role cannot be excluded and should be considered in similar clinical scenarios, especially in populations with a higher prevalence of intracranial atherosclerosis.

Initial evaluation should begin with a non-contrast CT scan to exclude intracranial hemorrhage. Once a hemorrhagic event has been ruled out, an MRI is recommended, as it provides superior anatomic detail and allows for more precise localization of ischemic injury. Among MRI techniques, DWI remains the gold standard for confirming the diagnosis. However, it is noteworthy that up to 30% of patients may not demonstrate an identifiable lesion on DWI, which can contribute to diagnostic uncertainty and potential misclassification of the syndrome [7]. In terms of prognosis, Wallenberg syndrome typically has more favorable functional outcomes compared with many other acute ischemic stroke subtypes. Nevertheless, residual disability is common, the most frequent being chronic gait instability. Early initiation of multidisciplinary rehabilitation, including physical and occupational therapy, is essential for optimizing functional recovery and improving long-term quality of life.

However, in this case, the patient's only presenting complaints were headache, nausea, and vomiting. This general and atypical presentation of the stroke did not automatically ring alarm bells, which suggested a stroke. The real presentation came afterward when she had collapsed on the hospital grounds, where she had presented with a left frontal headache radiating to the eye, as well as ataxia and chest pain with blurred vision. The CT head had not been helpful in the aid of diagnosing the stroke, but in fact, the MRI with DWI (Figure 1) and T2 FLAIR (Figure 2) had shown the stroke on the left side of the medulla oblongata. Her atypical presentation to the hospital then became more evident with the ataxia and the limb weakness. The ipsilateral weakness in this patient is a rare finding; however, it may be due to damage to the corticospinal tract as it passes through the medulla. The corticospinal tract controls voluntary movements and crosses to the opposite side of the body at the medulla. In this case, due to the stroke involving the post-decussation pyramidal tract in the medulla, she had ipsilateral weakness. The use of aspirin and clopidogrel is the antiplatelet regimen that is most commonly used to help prevent and reduce the risk of another stroke from occurring. In this case, the patient could not be thrombolysed, as she was outside the thrombolysis window, which can play an important role in the severity of the stroke. The later diagnosis of Horner's syndrome put into perspective the different signs and symptoms that can come with the disorder.

DAPT was initiated within 24 hours of symptom onset, in accordance with current guidelines for minor ischemic stroke or high-risk transient ischemic attack. DAPT was maintained for 21 days before transitioning to single antiplatelet therapy, balancing the benefit of early recurrence prevention with the risk of hemorrhagic complications.

## Conclusions

This case underscores the importance of recognizing that nonspecific symptoms such as headache, nausea, or vomiting can be early indicators of posterior circulation ischemia. These symptoms are often attributed to benign peripheral or gastrointestinal causes, which may delay the diagnosis of conditions like Wallenberg syndrome. When initial CT imaging is inconclusive, early MRI, particularly DWI, should be pursued to confirm brainstem involvement. Integrating this diagnostic vigilance into clinical practice is essential to avoid missed or delayed recognition of posterior circulation strokes and to initiate timely, evidence-based management.
